# Black People Living with Memory Challenges & Study Partners’ Preferred Activity Tracker Features

**DOI:** 10.1093/geroni/igaf122.3175

**Published:** 2025-12-31

**Authors:** Rebecca Lassell, Christopher Carey, Lola Sample, Hanley Elftmann, Laura Gitlin, Jaroslaw Harezlak, Richard Holden, NiCole Keith

**Affiliations:** Indiana University Bloomington, Bloomington, Indiana, United States; Indiana University School of Medicine, Indianapolis, Indiana, United States; Indiana University School of Public Health Bloomington, Bloomington, Indiana, United States; A.T. Still University School of Osteopathic Medicine, Mesa, Arizona, United States; Drexel University, Philadelphia, Pennsylvania, United States; Indiana University Bloomington, Bloomington, Indiana, United States; Indiana University Bloomington, Bloomington, Indiana, United States; Indiana University School of Public Health Bloomington, Bloomington, Indiana, United States

## Abstract

Activity tracker preferences of Black people living with memory challenges (PLMC) or dementia and study partners are largely unknown, which can impact wear-time adherence. We sought to identify Black PLMC and study partners’ preferred activity tracker features. Participants (n = 10) were recruited from Eskenazi Health in Indianapolis, Indiana. Data were collected through two 90-minute focus groups with Black PLMC participants (n = 5) and Black study partners (n = 5), respectively, and a PLMC and study partner interview. The focus group was guided by a semi-structured questionnaire and involved interacting with an Apple Watch SE, Oura Ring Heritage, Fitbit Inspire 3, and Polar Watch Ignite. Participants ranked each device based on comfort (device size, weight, material), convenience (battery life, water resistance, style, emergency button), and features (e.g., activity, steps, time standing, calories, distance/GPS, heart rate, respiratory rate) from 1-5 using checklists and picture cards. Focus groups were audio recorded. Device rankings from each category (comfort, convenience, and features) were summarized with descriptive statistics. Audio recordings were analyzed using the Rapid Identification of Themes from Audio recordings method and transcribed. PLMC rated the Apple Watch SE with mean scores in comfort (4.00), convenience (3.33), and features (4.3),30) emphasizing screen size, viewing progress on the watch, and an emergency button. Study partners rated the Fitbit Inspire 5 most in comfort (5), while the Apple Watch SE had the most rankings for convenience (4.75) and features (5.00), highlighting the emergency button and GPS as key attributes. Findings can guide activity tracker selection in future studies with this population.

